# 12. Modeled Impact of the COVID-19 Pandemic and Associated Reduced Adult Vaccinations on Herpes Zoster in the United States

**DOI:** 10.1093/ofid/ofab466.214

**Published:** 2021-12-04

**Authors:** Elizabeth M La, Desmond Curran, Ahmed Salem, David Singer, Nicolas Lecrenier, Sara Poston

**Affiliations:** GSK, Philadelphia, Pennsylvania

## Abstract

**Background:**

During the COVID-19 pandemic, adult vaccination in the United States (US) decreased substantially in 2020. Unlike other vaccine-preventable diseases where individuals may have experienced reduced risk due to COVID-related mitigation efforts (e.g., lockdown restrictions, use of face masks), individuals remained at risk of herpes zoster (HZ). This study projects the impact of reduced recombinant zoster vaccine (RZV) use on HZ cases and complications in the US.

**Methods:**

A multi-cohort Markov model estimated the impact of missed RZV vaccinations, by comparing scenarios with and without missed vaccinations between Apr-Dec 2020, on cases of HZ, postherpetic neuralgia (PHN), and quality-adjusted life-years (QALYs) among US adults aged ≥ 50 years. Epidemiology, RZV efficacy, and utility inputs were obtained from standard US sources, clinical trial data, and published literature. Missed doses were estimated using data on RZV doses and an assumed 43% reduction in RZV vaccinations during the pandemic, based on publicly available data. Deterministic sensitivity and scenario analyses were conducted.

**Results:**

In 2020, approximately 21 million (M) RZV distributed doses were expected, including an estimated 9.2M RZV series initiations in Apr-Dec. An estimated 3.9M RZV series initiations were missed, resulting in 31,945 projected HZ cases, 2,714 PHN cases, and 610 lost QALYs projected over a 1-year follow up. If individuals with missed RZV initiations remain unvaccinated in 2021, avoidable HZ cases will increase to 63,117 over 2 years. Further, if the same number of RZV initiations are missed in 2021, 95,062 avoidable HZ cases are expected. In a sensitivity analysis assuming 30% RZV reduction, 18,020 avoidable HZ cases and 1,531 PHN cases were observed over 1 year.

**Conclusion:**

Adding to the substantial COVID-19 infection-related morbidity and mortality, reduced RZV use during the pandemic resulted in further burden from avoidable HZ cases. Health care providers should continue to emphasize the importance of vaccination against HZ and other preventable diseases during the pandemic.

**Funding:**

GlaxoSmithKline Biologicals SA (GSK study identifier: [VEO-000222]).

**Acknowledgement:**

Business & Decision Life Sciences c/o GSK (Coordination: Quentin Rayée).

**Disclosures:**

**Elizabeth M. La, PhD**, **The GSK group of companies** (Employee, Shareholder) **Desmond Curran, PhD**, **The GSK group of companies** (Employee, Shareholder) **Ahmed Salem, MSc**, **The GSK group of companies** (Employee) **David Singer, PharmD, MS**, **The GSK group of companies** (Employee) **Nicolas Lecrenier, Ing, PhD**, **The GSK group of companies** (Employee, Shareholder) **Sara Poston, PharmD**, **The GSK group of companies** (Employee, Shareholder)

